# Patient initiated follow-up in cancer patients: A systematic review

**DOI:** 10.3389/fonc.2022.954854

**Published:** 2022-10-13

**Authors:** Claire Newton, Kinta Beaver, Andrew Clegg

**Affiliations:** ^1^ University Hospitals Bristol and Weston National Health Service (NHS) Foundation Trust, St. Michaels Hospital, Bristol, United Kingdom; ^2^ University of Bristol, Senate House, Bristol, United Kingdom; ^3^ School of Sport & Health Sciences, Faculty of Health & Wellbeing, University of Central Lancashire, Preston, United Kingdom; ^4^ Synthesis, Economic Evaluation and Decision Science (SEEDS) Group, Applied Health Research Hub, University of Central Lancashire, Preston, United Kingdom

**Keywords:** patient initiated follow-up (PIFU), oncology, survival, follow up, systematic review

## Abstract

**Background:**

Patient-initiated follow-up (PIFU) is increasingly being implemented for oncology patients, particularly during the COVID-19 pandemic, given the necessary reduction in face-to-face hospital outpatient appointments. We do not know if PIFU has a positive (or negative) impact on overall, or progression free, survival.

**Objectives:**

To investigate the impact of PIFU on overall survival, progression free survival, patient satisfaction, psychological morbidity, specifically quality of life (QoL) and economic costs compared to hospital follow up (HFU), for any type of cancer.

**Methods:**

We carried out a systematic review using five electronic databases: MEDLINE, CINAHL, EMBASE, PsycInfo and Cochrane Central Register of Controlled Trials. Studies were eligible if they were controlled clinical trials comparing PIFU with another form of active follow-up. Effectiveness was assessed using the primary outcome of overall survival and secondary outcomes of progression free survival, patient satisfaction, psychological morbidity, QoL and cost effectiveness.

**Results:**

Eight studies met the inclusion criteria and were included. Only one study included survival as a primary outcome and indicated no significant differences between hospital-based follow-up and PIFU, although not adequately powered to detect a difference in survival. For secondary outcomes, few differences were found between PIFU and other forms of active follow-up. One study reported significant differences in fear of cancer recurrence between PIFU and HFU although did not reach the limit of clinical significance; in the short term, fear decreased significantly more in hospital based follow-up.

**Conclusion:**

We do not have evidence to support the impact of PIFU on survival or progression free survival. Fully powered randomized controlled trials are required to determine the full impact of PIFU in the longer term.

## Introduction

Historically, patients diagnosed and treated for cancer were followed up in hospital outpatient clinics for 5-10 years following their original diagnosis (1–3); primarily to detect recurrences at an early stage and improve survival. There is little evidence that this approach improves survival (4, 5). However, there is evidence that hospital follow-up (HFU) does not meet the long-term physical, psychological and social needs of cancer survivors (6–8).

New approaches to cancer follow-up have been advocated with a shift away from searching for signs of recurrent disease to meeting the individual needs of patients (9). The Living With and Beyond Cancer program in the United Kingdom (UK) has reported that patients diagnosed and treated for cancer face many long-term physical and psychological challenges related to diagnosis and treatment and need continued support and information (9), with a prominent focus on supported self-management (10). Alternative strategies have been evaluated including General Practitioner (GP) follow-up, nurse-led, and telephone follow-up (11–13). Patient-initiated approaches have also been promoted, intended to provide patients with support mechanisms for access to specialist-based services but not requiring regular scheduled hospital appointments with a health care professional (14). In patient-initiated follow-up (PIFU) patients are not given routine follow-up appointments, but instead are asked to telephone a designated contact in the hospital (usually a clinical nurse specialist) if they have any pre-determined symptoms. There has been an increase in PIFU practices, particularly in gynecological oncology (15, 16) and other specialties such as breast and colorectal cancer where screening for recurrences exists (17).

During the COVID-19 pandemic many hospital-based appointments have been cancelled and a reliance on alternative approaches using modern technology has been evident for maintaining cancer follow-up services. For example, there have been recommendations to reduce hospital-based contact, and therefore transmission of COVID-19, for patients receiving follow-up during radiotherapy treatment, advocating remote monitoring by telemedicine or telephone calls (18). Cancer patients are at high risk for COVID 19 due to the risk factors of age, co-morbidities, immunosuppressed state and regular hospital visits (19). In Italy, one of the hardest hit countries in the early stages of the pandemic, follow-up appointments were delayed, and symptom focused follow-up and telemedicine approaches were recommended for patients ‘off treatment’ (20). A large survey of cancer patients in the Netherlands reported that the most frequently experienced impact of COVID 19 was a shift from HFU to contact by telephone (21). The UK’s National Institute for Health and Care Excellence (NICE) issued COVID 19 guidance on communicating with patients, recommending minimizing non-essential face-to-face contact (22). Although the easing of restrictions over time should allow for face-to-face appointments to be resumed, it is likely that follow-up appointments will increasingly encompass remote monitoring and patient-initiated approaches.

As yet, we do not know the full impact of remote monitoring on survival outcomes. A preliminary literature search indicated that clinical trials comparing PIFU with standardized practice (HFU) tended to focus on psychological outcomes such as psychological morbidity and quality of life (QoL) rather than survival (23, 24). Hence, the primary aim of this study was to systematically review published studies for evidence of the impact of PIFU on overall survival, for any type of cancer. Our secondary aims were to investigate if there were any differences in progression free survival, patient satisfaction, psychological morbidity, QoL and economic costs between PIFU and other active forms of follow-up (e.g. HFU, GP follow-up, nurse-led follow-up, telephone follow-up).

## Methods

Our systematic review followed a protocol adhering to recognized guidance and reporting standards (see **Additional File 1** for PRISMA checklist) (25, 26). We identified studies through searches of five electronic databases, specifically MEDLINE, CINAHL, EMBASE, PsycInfo and Cochrane Central Register of Controlled Trials (see **Additional File 2** for search strategy). All databases were searched from their inception to November 2020 and were limited to studies published with an English language abstract. Additional references were identified through screening reference lists of included studies and relevant systematic reviews.

Studies were eligible if they were controlled clinical trials comparing any form of PIFU with another form of active follow-up (including different forms of PIFU). Participants were people aged 18 years or over with any cancer diagnosis. Effectiveness was assessed using the primary outcome of overall survival and secondary outcomes of progression free survival, patient satisfaction, psychological morbidity, QoL and cost effectiveness (e.g. cost per quality adjusted life years). Studies were excluded if participants were actively receiving treatment (including palliative treatment), except hormonal treatment, bevacizumab, PARP inhibitors or other maintenance treatments. Studies were also excluded if patients had reported side-effects (e.g. treatment toxicities) as they would not be eligible for PIFU. Interventions using only telephone or HFU were also excluded as telephone is not a form of PIFU. Abstracts and conference proceedings were only considered if enough detail of their methodology and results were published.

Studies were selected through two stages. First, titles and abstracts were screened using pre-specified and piloted criteria, with manuscripts of studies appearing to meet the criteria retrieved and assessed at a second stage. Data was extracted using a pre-piloted form and included characteristics of PIFU and comparator, setting, participants characteristics, outcomes assessed, and study funding. When further information was required, attempts were made to contact the authors for clarification. Risk of bias was assessed using the Cochrane Collaboration risk of bias tool (25). All stages in study selection, data extraction and risk of bias were undertaken independently by two reviewers, with any disagreements resolved through discussion or arbitration by an independent third reviewer.

Studies were synthesized through a narrative synthesis with tabulation of results of included studies. Studies were not pooled through meta-analyses due to heterogeneity among the studies, particularly in the participants, interventions and outcomes reported.

## Results

**Figure 1:**
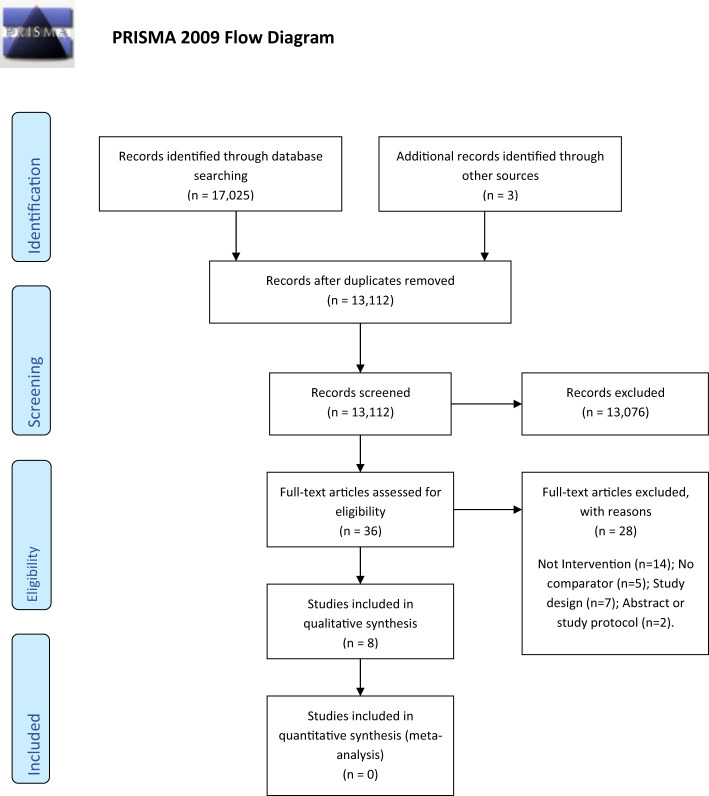
PRISMA flow diagram.

The search strategy identified 17,028 papers which, after duplicate removal, resulted in 13,112 papers for inspection. Screening of titles and abstracts excluded 13,078 records (**Figure 1**). Manuscripts for 36 papers were screened, with eight studies included in the review, representing four cancer types (breast n=4, colorectal n=2, endometrial n=1, prostate n=1). There were six randomized controlled studies; four were multi-center. The remaining two studies used historical or non-randomized comparisons. See **Table 1** for a summary of included studies.

**Table 1:** Summary of included studies.

Author (year)Country	Type of Cancer	Study design (number of centers)	Inclusion criteria	Intervention (number of participants)	Comparator (number of participants)	Primary outcome	Length of follow up (months)
Frankland et al. (2019) (27) UK	Prostate	Service evaluation (historical control group) using validated questionnaires	Primary treatment – radical prostatectomy, radiotherapy or primary androgen deprivation therapy	PIFU (n= 293)	HFU (n= 334)	Quality of life Unmet survivorship needs	8
Jeppesen et al. (2018) (23) Denmark	Endometrial	RCT (4) using validated questionnaires	Stage 1 -grade 1/2	PIFU (n= 105)	HFU (n= 107)	Fear of cancer recurrence	36
Batehup et al. (2017) (28) UK	Colorectal	Service Evaluation. Non-randomised comparison using validated questionnaires	post curative surgery	PIFU + 3 monthly stool samples yrs1-2, colonoscopy + self-management program (n=239)	HFU + stool samples, colonoscopy (n= 124)	Quality of life	12
Kirshbaum et al., 2016) (24) UK	Breast	RCT (1) using validated questionnaires	Stage 1 or 2	PIFU + educational program (n= 56)	HFU (n= 56)	Quality of life	24
Sheppard et al. (2009) UK	Breast	RCT (1) using validated questionnaires	2 years post treatment	PIFU + annual mammogram (n= 112)	HFU + annual mammogram (n= 112)	Quality of life	18
Koinberg et al. (2004) (29) Sweden	Breast	RCT (2) using validated questionnaires	Stage 1 or 2	PIFU + annual mammogram + education (n= 133)	HFU + annual mammogram (n= 131)	Hospital anxiety and depression	60
Brown et al. (2002) (30) UK	Breast	RCT (2) using validated questionnaires and structured interviews	Stage 1	PIFU + annual mammogram (n= 30)	HFU + annual mammogram (n= 31)	Quality of life and psychological morbidity	12
Ohlsson et al. (1995) (31) Sweden	Colorectal	RCT (2)	3 months post curative surgery	PIFU + 3 monthly stool samples yrs1-2 (n= 53)	Intensive HFU + colonoscopy (n= 53)	Disease free survival	81

PIFU, patient initiated follow up; HFU, hospital follow up; RCT, randomized controlled trial.

Assessment of the risk of bias showed that the reliability of the results was uncertain due to the variability in the methodological rigor of the studies and, as such, the findings should be interpreted with caution (**Additional File 3**). Of the eight studies, seven studies had four or more criteria judged unclear or at high risk of bias (23, 24, 27, 28, 30–32), with only one study having five criteria assessed at low risk (29). Importantly, only three studies were considered to have a low risk of bias on the key criteria of random sequence generation and allocation concealment (23, 29, 32), with all other studies at high or uncertain risk of selection bias. Given the nature of the interventions, blinding of participants and of those delivering interventions was unlikely, reflected in none of the studies being judged at low risk. In addition, only one study was considered to have a low risk of bias on blinding of those assessing outcomes (23). The lack of blinding indicates that all studies were at some risk of both performance and detection bias.

### Overall survival

Only one study with 106 participants followed up after curative resection of colorectal cancer investigated the primary endpoint of overall survival (31). However, this study was not adequately powered; 1000 patients would have been required to detect a 9% difference in five year survival (31). There were 18 patients versus 17 patients with recurrent disease in the PIFU and hospital follow up (HFU) groups respectively. Re-resection with curative intent was performed in only three patients in the PIFU group and in five patients (four were asymptomatic) in the HFU group. The five-year survival rate for colorectal cancer was 67% in the PIFU group and 75% in the HFU group (P > 0.05); the corresponding cancer-specific survival rates were 71% and 78% respectively (31).

### Tumor recurrence and fear of cancer recurrence

There were five studies (three breast, one endometrial, one colorectal) (23, 29–32) that reported recurrent disease but all five studies had relatively low numbers of participants (range 61 – 627 participants) and were not adequately powered to detect a difference between PIFU and HFU with regard to recurrence rates. This was because recurrence was not their primary outcome measure. Overall, there was no difference between recurrent disease between PIFU and HFU and numbers of recurrences were low (see **Additional File 4**).

There were three studies (one breast, one endometrial, one prostate) (23, 27, 32) that reported on fear of cancer recurrence (FCR); each used a different measurement tool (see **Additional File 4**). Only one study reported significant differences in FCR between PIFU and HFU with improvement in FCR scores from baseline to 10 months follow-up of 8.0 *vs.* 1.4 for the HFU and PIFU groups respectively (23). FCR decreased significantly more in the HFU group with an estimated difference of -5.9 (95% CI: [-10.9; -0.9], p= 0.02). However, the proportion of women with clinical FCR did not differ between the groups at 10-months (odds ratio= 0.9, (95% CI: [0.32; 2.67], p= 0.89). Overall, 20% were struggling with FCR at 10 months post treatment regardless of type of follow-up (23).

### Health care use and economic evaluation

Two studies had carried out economic evaluations (27, 28). In a colorectal cancer study, PIFU was £142.24 per patient more expensive than HFU in the first year, due primarily to an additional self-management workshop (28). However, PIFU saved patients £28.38 per year for travel costs. In a prostate cancer study, the direct costs of the PIFU approach were £102 per patient compared to £59 per patient in the HFU group, again due to the cost of a patient focused workshop at £63 per participant (27). When direct costs and costs of service use were combined, the PIFU group had lower overall average costs of £289 per patient vs. £327 for HFU (27).

Four studies reported health care use in terms of additional visits to GP’s and number of telephone calls to specialist services (23, 28, 30, 32). There were no significant differences in health care use between PIFU and HFU in two studies that included breast cancer patients (30, 32). In an endometrial cancer study there was a slight increase in cancer-related GP visits in the PIFU vs. HFU groups (213 vs. 135, p=0.77), but there was a large reduction in hospital appointments in the PIFU group (19 vs. 139,p<0.01) (23). A non-randomised study showed GP visits were higher for hospital based follow up than PIFU patients (1.84 vs. 1.08 monthly visits, p = 0.024) in colorectal cancer patients (28).

### Patient satisfaction

Four studies included outcome data on patient satisfaction. Two studies, involving breast cancer participants, reported no significant differences in patient satisfaction between PIFU and HFU (29, 30). However, Brown et al. (2002) reported convenience as an advantage at six months in PIFU vs. HFU respectively; 16/27 vs. 1/24 patients [chi2 17.354, p=0.000, df 1] which continued to 12 months; 22/27 vs. 1/24 patients [chi2 30.79, p=0.000, df 1] (30). In addition, more women reported reassurance as an advantage at 6 months in PIFU vs. HFU respectively; 1/27 vs. 18/20 patients [chi2 27.63, p=0.000, df 1] which continued to 12 months; 3/27 vs. 20/20 patients; [chi2 24.17, p=0.000, df 1] (30).

There were also no significant differences between PIFU and HFU with patient satisfaction in a study of 363 patients with colorectal cancer with respect to reassurance, access to specialist support, ability to ask questions, time spent with doctors/nurses, and involvement in decision-making (Mann Whitney U test p = 0.371) (28). However, more patients in the PIFU group reported their follow up as acceptable than patients on HFU; 36/37; 97.3% vs. 24/32 patients; 75%; p= 0.010 (28)

In a study that included 627 participants with prostate cancer, significantly more patients at four months in the PIFU group vs. HFU group were satisfied for 9 of 11 statements (p=0.015) (27). However, this difference did not last to eight months with only one statement ‘I have known who to contact with any problems’ showing more agreement for the PIFU group (27).

### Quality of life and psychological morbidity

Five studies reported on cancer specific QoL and/or overall QoL (24, 27, 28, 30, 32); two used the European Organisation for Research and Treatment of Cancer questionnaires (EORTC) and three used the Functional Assessment of Cancer Therapy (FACT) questionnaires. Five studies reported on psychological morbidity (24, 27, 29, 30, 32); two used the General Health Questionnaire (GHQ) and three used the Hospital Anxiety and Depression (HAD) scale.

There was no significant difference in cancer specific QoL or psychological morbidity (using the GHQ) between PIFU and HFU in 224 women with breast cancer over 18 months (32). However, there was a trend towards a favorable benefit of PIFU over HFU for the FACT- breast subscale, although this was not statistically significant adjusted mean (PIFU-HFU) -1.7 (95% CI: -3.2, 0.5), p=0.058 (32).

There were also no statistically significant differences in QoL or psychological morbidity (using the HAD scale) between PIFU and HFU in 61 patients with breast cancer except in the arm symptoms and breast symptoms subscales (30). Both these subscales had higher baselines in the HFU group, which continued to be higher (see **Additional File 5** for significant findings). This could be attributed to bias in blinding, allocation bias and attrition bias in this randomized controlled trial (RCT).

There was no significant difference in psychological morbidity (using the HAD scale) between PIFU and HFU in two studies of breast cancer over 2-5 years (24, 29). Overall, levels of anxiety and depression were low, ranging between 4.4% -11.6% and between 0.8% -5.2% for anxiety and depression respectively.

In a study of 363 patients with colorectal cancer there was statistically significant better QoL in the PIFU group compared to HFU group (see **Additional File 5**) using FACT and EQ-5D-L questionnaires (28). There was also a significant improvement in mental health (p=0.032), unmet needs (−2.4 [95% CI −4.5, −0.3] p= 0.025), and total unmet needs (−1.2 [95% C.I −2.3, −0.2] p= 0.02) in the PIFU group compared to HFU at 4 months but none of these were significant at 8 months (28).

## Discussion

This systematic review primarily examined the evidence for the impact of PIFU on overall survival for patients diagnosed and treated for any type of cancer. We found very little evidence in this area. We included data from eight studies; half of these related to breast cancer patients. Only six studies were randomized controlled designs and only one study, involving 106 colorectal cancer patients dating back to 1995, investigated disease free survival as a primary outcome although it was not adequately powered to detect this (31). However, despite this lack of evidence, PIFU is strongly advocated, especially during the COVID-19 pandemic. A recently published document by the National Health Service (NHS) in England recommended PIFU for a wide range of patients, including oncology patients (33). Benefits to patients, clinicians and organizations were presented. Supporting references were provided but none of the references report on an RCT that compares hospital-based follow-up with PIFU with survival as a primary outcome, for any disease type. More research is clearly needed in this area to determine if PIFU impacts on overall, and progression-free, survival.

Although only one study in this review investigated survival as the primary outcome, there was some exploration of tumor recurrence rates and fear of recurrence. Unfortunately, studies were not adequately powered to detect a difference between PIFU and HFU with regard to recurrence rates but findings tended to indicate no significant differences. Three studies had explored FCR, with conflicting findings. Two studies reported no differences between groups (27, 32), although one RCT did indicate that hospital-based follow-up was more likely to alleviate FCR although the threshold for clinical significance was not reached (23). FCR is a major concern for patients and one of the most common unmet needs (34), regardless of cancer type (35). It has been reported that FCR is negatively correlated with information provision (35). In this case, it is vital that patients who are allocated PIFU as a follow-up strategy are well informed and have access to the information they need to self-manage their condition. There is a clear distinction between ‘no follow-up’, with an expectation that patients will initiate contact if they have any concerns, and supported self-management approaches that provide patients with information and support mechanisms (36).

It may have been anticipated that health care costs would be lower for PIFU but this was not necessarily the case. Ensuring that patients were well informed and well prepared to self-manage their condition could encompass costly educational events in the short term (28). However, over time, PIFU was not more costly to the health services and patients saved time and travel expenses. The NHS in England intends to transform outpatient services to avoid up to a third of face-to-face outpatient visits, removing the need for up to 30 million outpatient appointments a year, with substantial savings on health care costs (37). This intention was stated prior to the COVID-19 pandemic and is part of the personalized care agenda where patients are encouraged to manage their own condition and take responsibility for their health and wellbeing.

PIFU will have a vital role to play in reducing outpatient appointments and promoting supported self-management models of care. Hence, it is encouraging that our review indicated that, in general, patients were equally satisfied with HFU and PIFU. Those allocated to PIFU tended to indicate the convenience of the approach, while those in HFU tended to be more reassured by face to face appointments (30). Patients were more likely to know who to contact if allocated to PIFU (27). An identifiable point of contact has been strongly advocated in self-management approaches, gaining access back to specialist services with minimal delay. A recent study involving 228 women, allocated to PIFU following treatment for endometrial cancer, reported that approximately 20% of participants contacted a clinical nurse specialist at least once during the study period (38). Patient initiated contact was more likely in the first six months, with contact being primarily related to physical problems but also to a need for psychological support (38). Therefore, clear processes for making contact with, and accessing, specialist services, will be essential in implementing PIFU across a range of diagnoses and disease conditions.

We found little evidence of PIFU having a negative impact on psychological morbidity or QoL. One study indicated that mental health and QoL was better in the PIFU arm but this was of short duration and there were no differences apparent at the eight months timepoint (28). Levels of anxiety and depression tended to be low for both HFU and PIFU. A number of different measures had been used in the studies to measure psychological morbidity and both cancer specific QoL and generic QoL life. Hence, it was difficult to make meaningful comparisons. However, in general, study participants allocated to PIFU did not experience increased psychological morbidity or a reduction in quality of life. These are encouraging findings for the successful implementation of PIFU.

Regular assessment of both physical and psychological health (including psychological morbidity and QoL) has been recommended as part of any risk stratification strategy used to determine the most appropriate follow-up pathways for cancer patients (17). While our review findings support that many patients may be comfortable with PIFU and are well able to self-manage their own condition, given appropriate access routes back to specialist care, there are other patients who may not feel confident to self-manage and need regular assessments of physical, psychological and social needs. Although PIFU may be a very useful short-term strategy to reduce transmission of COVID-19, fully powered RCT’s are required as a matter of urgency to ensure we have the evidence to support the effective implementation of PIFU on a longer term basis and to be clear that PIFU does not have an adverse effect on overall or progression free survival or create unresolved physical and psychological morbidities. Recruiting patients to an RCT of HFU versus PIFU may be challenging, particularly if patients have already experienced the reassurance of hospital appointments. A recent feasibility study indicated that endometrial cancer patients may be reluctant to undergo randomization, although those who were randomized were highly satisfied with PIFU (36). However the TOTEM study demonstrated 1871 patients were needed to detect a 5% difference in overall survival between two follow up regimens for endometrial cancer was achievable. This study compared more intensive follow up to standard follow up and there was no difference in overall survival (39).

## Limitations

Our review was limited by the number of studies included, with a total of 1,969 participants across the eight included studies. Only four studies had been published since 2000 and the four that were published more than 10 years ago are arguably now out of date, given recent advances in promoting patient-initiated models of follow-up care. The studies only represented four cancer types, with half the studies carried out with breast cancer patients. In addition, there was a diversity of measures used to determine study outcomes and this meant we were not able to carry out meta-analysis.

## Conclusion

There is a strong shift away from hospital-based follow-up appointments in oncology, towards more patient centered approaches, including PIFU. During the COVID-19 pandemic this has been especially relevant and prevalent. However, our review indicates that, while we may have evidence that PIFU may not negatively impact patient satisfaction, psychological morbidity and QoL, we do not have evidence to support the impact of PIFU on survival or progression free survival. There are few economic evaluations in this area and PIFU may not necessarily equate to cost savings for health services, although the approach is likely to be convenient for patients and save travel costs. Fully powered RCT’s are required to determine the full impact of PIFU in the longer term with achievable sample sizes.

## Data availability statement

The original contributions presented in the study are included in the article/Supplementary Material. Further inquiries can be directed to the corresponding author.

## Author contributions

CN, KB, and AC conceptualized and designed the study. All authors were responsible for developing the search strategy in collaboration with information specialists; all authors contributed to data acquisition and quality control of data. All authors contributed to data extraction, analysis and drafting the manuscript. All authors have critically reviewed, edited and approved the final version of the manuscript.

## Acknowledgments

The authors would like to acknowledge the support of Janet Reed and Cath Harris (Information Specialists, University of Central Lancashire) who helped develop and run the searches strategy, and the library at University Hospitals Bristol and Weston NHS trust.

## Conflict of interest

The authors declare that the research was conducted in the absence of any commercial or financial relationships that could be construed as a potential conflict of interest.

## Publisher’s note

All claims expressed in this article are solely those of the authors and do not necessarily represent those of their affiliated organizations, or those of the publisher, the editors and the reviewers. Any product that may be evaluated in this article, or claim that may be made by its manufacturer, is not guaranteed or endorsed by the publisher.

## Author disclaimer

AC is part-funded through the National Institute for Health Research Applied Research Collaboration North West Coast (ARC NWC). The views expressed are those of the authors and not necessarily those of the National Institute for Health Research or the Department of Health and Social Care.

## Supplementary material

Additional file 1 Prisma checklist.

Additional file 2 Medline Search strategy.

Additional file 3 Risk of bias for included studies.

Additional file 4 Tumor recurrence and fear of cancer recurrence.

Additional file 5 Results of studies compared for quality of life and psychological morbidity.
