# Supplementary material for: Patient initiated follow-up in cancer patients: A systematic review
Source: Front Oncol. 2022 Oct 13;12:954854. doi: 10.3389/fonc.2022.954854 (PMC9606321; doi:10.3389/fonc.2022.954854)
Supplement: Additional file 2 — Medline Search strategy. [file Table_2.docx]

Additional file 2. Medline Search Strategy

| 1 | "Referral and Consultation"/ |
| --- | --- |
| 2 | Health Services Accessibility/ |
| 3 | open access.mp. |
| 4 | Aftercare.mp. or Aftercare/ |
| 5 | (follow-up* or follow up*).mp. |
| 6 | (clinic* or clinics*).mp. |
| 7 | (outpatient* or out patient*).mp. |
| 8 | (check up* or checkup or check ups* or checkups).mp. |
| 9 | (appointment* or hospital appointment*).mp. |
| 10 | surveillance.mp. |
| 11 | Self-Management/ or *Self Care/ |
| 12 | (self-management or self management or self-care or self care).mp. |
| 13 | monitoring.mp. |
| 14 | support.mp. |
| 15 | referral.mp. |
| 16 | psychol*.mp. |
| 17 | peer support.mp. |
| 18 | information.mp. |
| 19 | reassurance.mp. |
| 20 | satisfaction.mp. |
| 21 | communication/ |
| 22 | access*.mp. |
| 23 | patient education.mp. |
| 24 | PSA Surveillance.mp. |
| 25 | community follow-up.mp. |
| 26 | on demand or on-demand.mp. |
| 27 | outpatients/ |
| 28 | patient views.mp. |
| 29 | or/1-28 |
| 30 | patient-initiated.mp. |
| 31 | patient-led.mp. or Patient Satisfaction/ |
| 32 | (patient adj1 triggered).mp. |
| 33 | (patient adj1 request).mp. |
| 34 | (Patient* adj3 led adj5 (outpatient* or out patient*)).mp. |
| 35 | Or/30-34 |
| 36 | oncology.mp. |
| 37 | Neoplasms/ |
| 38 | cancer.mp. |
| 39 | Or/36-38 |
| 40 | survival/ |
| 41 | survival.mp. |
| 42 | "recurrence free survival".mp. |
| 43 | "overall survival".mp. |
| 44 | "time to detection of recurrence".mp. |
| 45 | detection.mp. |
| 46 | recurrence/ |
| 47 | recurrence.mp. |
| 48 | "Patient reported outcomes".mp. |
| 49 | Patient Reported Outcome Measures/ |
| 50 | PROMS.mp. |
| 51 | anxiety/ |
| 52 | pain/ |
| 53 | symptoms.mp. |
| 54 | complications.mp. |
| 55 | satisfaction.mp. |
| 56 | adverse events.mp. |
| 57 | QALY.mp. |
| 58 | Quality-adjusted life years/ |
| 59 | Quality adjusted life years.mp. |
| 60 | quality of life/ |
| 61 | or/40-60 |
| 62 | Clinical Trials as Topic/ |
| 63 | Randomized Controlled Trials as Topic/ |
| 64 | RCTs.mp. |
| 65 | (randomised trial or randomized trial).mp. |
| 66 | trial.mp. |
| 67 | Non-randomised controlled trials.mp. |
| 68 | quasi experimental studies.mp. |
| 69 | Non-Randomized Controlled Trials as Topic/ |
| 70 | clinical trial.mp. |
| 71 | (study or studies).mp. |
| 72 | (randomised or randomized).mp. |
| 73 | comparative stud*.mp. |
| 74 | Cost effectiveness studies.mp. |
| 75 | or/68-79 |
| 76 | 29 and 35 |
| 77 | 76 and 39 and 75 |
| 78 | 77 and 61 |
| 79 | limit 78 to (english language and "all adult (19 plus years)") |
