# Supplementary material for: Patient initiated follow-up in cancer patients: A systematic review
Source: Front Oncol. 2022 Oct 13;12:954854. doi: 10.3389/fonc.2022.954854 (PMC9606321; doi:10.3389/fonc.2022.954854)
Supplement: Additional file 3 — Risk of bias for included studies. [file Table_3.docx]

Additional file 3. Risk of bias for included studies

| **Author**  **(year)** | **Random sequence generation** | **Allocation concealment** | **Blinding (performance and detection) bias participants** | **Blinding (performance and detection bias) those delivering the intervention** | **Blinding (performance and detection) bias those assessing the outcome** | **Incomplete outcome data (attrition bias)** | **Selective reporting (reporting bias)** | **Other biases** |
| --- | --- | --- | --- | --- | --- | --- | --- | --- |
| Frankland et al. (2019) | High | High | High | High | High | Low | Low | low |
| Jeppesen et al. (2018) | Low | Low | High | High | Low | High | High | Low |
| Batehup et al. (2017) | High | High | High | High | High | High | Unclear | High |
| Kirshbaum et al.  (2016) | Unclear | Unclear | High | High | Unclear | High | Low | Unclear |
| Sheppard et al. (2009) | Low | Low | High | High | Unclear | High | Unclear | Low |
| Koinberg et al. (2004) | Low | Low | Unclear | Unclear | Unclear | Low | Low | Low |
| Brown et al. (2002) | Low | Unclear | Unclear | Unclear | Unclear | Unclear | Unclear | Unclear |
| Ohlsson et al. (1995) | Unclear | Unclear | Unclear | Unclear | Unclear | Low | Low | Low |
