# Supplementary material for: Patient initiated follow-up in cancer patients: A systematic review
Source: Front Oncol. 2022 Oct 13;12:954854. doi: 10.3389/fonc.2022.954854 (PMC9606321; doi:10.3389/fonc.2022.954854)
Supplement: Additional file 4 — Tumor recurrence and fear of cancer recurrence. [file Table_4.docx]

Additional File 4. Tumour recurrence and fear of cancer recurrence

| **Author**  **(year)** | **Type of cancer** | **Tumour recurrence number of patients**  **PIFU vs HFU** | **Fear of cancer recurrence (FCR)**  **PIFU vs HFU**  **(Standard deviation)** |
| --- | --- | --- | --- |
| Frankland et al. (2019) | Prostate | N/A | **Worry of Cancer Scale (2 items, scores range from 0-20, higher score indicates greater worry)**  Baseline: 6.9 (4.4) vs 7.2 (5.0)  4 months: 6.3 (4.5) vs 6.1 (4.8) p= 0.224.  8 months:6.0 (4.4) vs 6.1 (4.7) p=0.246 |
| Jeppesen et al. (2018) | Endometrial | 0 vs 2 | **Fear of Cancer Inventory (42 items, 7 subscales, scores range from 0 -168, higher score indicates higher levels of FCR)**  Baseline: 44.8 (27.3) vs 48.5 (28.8)  10 months: 43.4 (26.5) vs 40.5 (28.5)  % decrease FCR -5.9 (95% CI: [-10.9; -0.9], p= 0.02  % with clinical FCR: OR 0.9 (95% CI: 0.32; 2.67) p=0.89.  no difference at 10 months 22.1% vs 17.7% |
| Sheppard et al. (2009) | Breast | 5 vs 4 | **Fear of Recurrence questionnaire (3 items, 5 point likert scale, higher scores indicate higher levels of fear)**  Baseline: 5.8 vs 6.0;  9 months: 5.6 vs 5.7  18 months: 5.6 vs 5.0 [adjusted mean 0.5 (95% CI: -0.3, 1.0)] p=0.066 |
| Koinberg et al. (2004) | Breast | 12 vs. 8 loco-regional recurrences  9 vs 9 distant metastases | N/A |
| Brown et al. (2002) | Breast | 2 vs 2 | N/A |
| Ohlsson et al. (1995) | Colorectal | 18 vs 17 | N/A |

PIFU=patient initiated follow up, HFU= hospital follow up. IQR= interquartile range.
