# Supplementary material for: Patient initiated follow-up in cancer patients: A systematic review
Source: Front Oncol. 2022 Oct 13;12:954854. doi: 10.3389/fonc.2022.954854 (PMC9606321; doi:10.3389/fonc.2022.954854)
Supplement: Additional file 5 — Results of studies compared for quality of life and psychological morbidity. [file Table_5.docx]

Additional file 5. Results of studies compared for quality of life and psychological morbidity.

| **Author**  **(year)** | **Type of cancer** | **Health-related QoL**  **(PIFU vs HFU)** | **Cancer-specific QoL**  **(PIFU vs HFU)** | **Psychological well being**  **(PIFU vs HFU)** |
| --- | --- | --- | --- | --- |
| Frankland et al. (2019) | Prostate | **CaSUN** (higher score = more unmet needs)  Total score  4 months:  −2.4 (95% CI −4.5, −0.3) p= 0.025  8 months:  −1.7 (95% C.I −3.7, 0.3) p=0.106  Total number unmet needs  4 months:  −1.2 (95% C.I −2.3, −0.2) p= 0.02  8 months:  −0.9 (95% C.I −2.0 - 0.1) n=517 p= 0.097 | **FACT-G** total mean [SD]:  4 months: 89.2 (13.1) vs 87.4 [14] p=0.96;  8 months: 88.4 (14.2) vs 87.2 (14.6)  p= 0.849. | **GHQ 12** (SD) higher score = worse  Baseline:  (2.1) vs 1.3 (2.4)  4 months:  0.9 (1.9) vs 1.3 (2.4)  -0.3 (-0.6, -0.03) p=0.032  8 months:  0.9 (2.0) vs 1.1 (2.3)  -0.08 (-0.3, 0.2) p=0.583 |
| Batehup et al. (2017) | Colorectal | **EQ-5D-5L** mean [SD]: 0.87 [0.15] vs 0.79 [0.16] p=0.033.  **EQ5D VAS** mean [SD]: 83.07 [13.07] vs 73.57[16.66] p=0.006 | **FACT-G** total mean [SD]:  91.15 [12.56] vs 85.48 [13.58] P=0.063.  **FACT-C** mean [SD]:  23.55 [3.05] vs 21.77 [3.75] p=0.026. | N/A |
| Kirshbaum et al. (2016) | Breast | **QLQ- C30**  Subscales at 24 months (SD):  QL2 Global health status 69.9(20.5) vs 75 (19.8), RF2 Role functioning 82.2 (22.7) vs 85.6 (23.6), EF emotional functioning 75.3 (24.8) vs 75.4 (27.4), CF Cognitive functioning 76.9 (21.4) vs 80.3 (24.4), Social functioning 86.6 (20.8) vs 85.5 (21.7), Fatigue 27.2 (24.6) vs 30.2 (26.9), Financia difficulties 5.7 (21.9) vs 10.3 (27.7) | **EORTC QLQ- BR23**  Subscales at 24 months (SD)  Body image 79.4 (25.9) vs 80.4 (26.2), Sexual functioning 24.7 (25.0) vs 18.4 (23.7), sexual enjoyment 61.1 (25.1) vs 72.7 (32.7), future perspectives 55.2 (30.1) vs 58.3 (31.2), systemic therapy side effects 26.4 (20.7) vs 25.5 (22.4), Breast symptoms 11.5 (12.8) vs 9.3 (11.2), Arm symptoms 12.0 (15.0) vs 15.4 (20.8) | **HAD scale**  **Depression** mean (SD):  Baseline: 3.19 (2.72) vs 3.53 (3.45)  6 months: 2.99 (2.89) vs 3.58 (3.70)  24 months: 3.71 (3.24) vs 3.94 (4.12)  **Anxiety** mean (SD):  Baseline: 6.59 (3.42) vs 5.71 (4.43)  6 months: 6.39 (4.05) vs 5.53 (4.12)  24 months: 7.00(4.60) vs 5.74 (5.00) |
| Sheppard et al. (2009) | Breast | N/A | n=107 each group  **FACT-G:**  adjusted mean (PIFU-HFU) 0.1 (95% CI: -3.2, 3.4), p=0.952.  **FACT - breast subscale:**  adjusted mean (PIFU-hospital FU) -1.7 (95% CI: -3.2, 0.5), p=0.058;.  **FACT-endocrine subscale**: adjusted mean (PIFU-HFU) -1.2 (95% CI: -4.2, 1.6), p=0.388.  **FACT-B**: adjusted mean (PIFU-HFU) -1.6 (95% CI: -4.6, 8.0), p=0.282]. | n=107 each group  **GHQ**  adjusted mean (PIFU-HFU) -0.1 (95% CI: -1.4, 1.0) p=0.767  **Fear**  Adjusted mean (PIFU-HFU)  0.5 (95% CI:0.3 to 1.0) p= 0.066 |
| Koinberg et al. (2004) | Breast | N/A | N/A | **HAD Scale:**  **Anxiety** –  6 months: RR1.8 (95%C.I 0.7-4.8)  60 months RR1.8 (95%C.I 0.6-5.1)  **Depression**  6 months: RR1.0 (95%C.I 0.6-16.4)  60 months RR 1.7 (95%C.I 0.4-7.2) |
| Brown et al. (2002) | Breast | **EORTC QLQ-C30**  At 12 months (IQR)  Physical scales 8(1) vs 8(1), Cognitive scales 4(1) vs 6(1), emotional scales 15(3) vs 15(3.5), social scales 4(0) vs 5(0.25), global quality of life scales 14(2) vs 14(3) | **EORTC QLQ-BR23**:  arm symptoms at baseline  3 vs 3.5 (U:272, p=0.003)  6 months:  3 vs 4 (U:271, p=0.028)  breast symptoms at baseline  4 vs 5 (U:309.5, p=0.033)  12 months:  4 vs 5 (U: 257.5, P=0.024) | **HAD Scale:**  **Anxiety** at baseline  (U: 362, p=0.135),  6 months: (U: 315.5, p=0.207),  12 months: (U:281.5, p=0.069)  **Depression** at baseline  (U 368, P=0.147),  6 months: (U:354.5, p=0.529),  12 months: (U:321, p=0.232). |

QoL: Quality of Life, HFU: hospital follow-up, PIFU: patient-initiated follow-up, GHQ: General Health Questionnaire, CaSUN: Cancer Survivors Unmet Needs, HAD: Hospital Anxiety and Depression scale.
